# Supplementary material for: SARS-CoV-2 Spike Protein Intensifies Cerebrovascular Complications in Diabetic hACE2 Mice through RAAS and TLR Signaling Activation
Source: Int J Mol Sci. 2023 Nov 16;24(22):16394. doi: 10.3390/ijms242216394 (PMC10671133; doi:10.3390/ijms242216394)
Supplement: Supplementary file 1 [file ijms-24-16394-s001.zip › ijms-2683498-supplementary.pdf]

Table S1. Table of Primers.

Gene	Species	FWD sequence	REV sequence
ACE-2	Mouse	5′-TCC ATT GGT CTT CTG CCA TCC G-3′	5′-AGA CCA TCC ACC TCC ACT TCT C-3′
AngII	Mouse	5′-GGT CAG TAC AGA CAG CAC CCT A-3′	5′-ACA CCG AGA TGC TGT TGT CCA C-3′
AT_1R	Mouse	5′-GCC ATT GTC CAC CCG ATG AAG T-3′	5′-ACA CAT TTC GGT GGA TGA CGG C-3′
AT_2R	Mouse	5′-CGT GAC CAA GTC CTG AAG ATG G-3′	5′-GGA AGT GCC AGG TCA ATG ATG ACT G-3′
GAPDH	Human	5′-GTC TCC TCT GAC TTC AAC AGC G-3′	5′-ACC ACC CTG TTG CTG TAG CCA A-3′
	Mouse	5′-CAT CAC TGC CAC CCA GAA GAC TG-3′	5′-ATG CCA GTG AGC TTC CCG TTC AG-3′
HMGB1	Mouse	5′-CCA AGA AGT GCT CAG AGA GGT G-3′	5′-GTC CTT GAA CTT CTT TTT GGT CTC-3′
Il-1β	Mouse	5′-TGG ACC TTC CAG GAT GAG GAC A-3′	5′-GTT CAT CTC GGA GCC TGT AGT G-3′
Il-6	Human	5′-AGA CAG CCA CTC ACC CTCT TAC G-3′	5′-TTC TGC CAG TGC CTC TTT GCT G-3′
	Mouse	5′-TAC CAC TTC ACA AGT CGG AGG C-3′	5′-CTG CAA GTG CAT CAT CGT TGT TC-3′
MasR	Mouse	5′-CTG ACA GCC ATC AGT GTG GAG A-3′	5′-GTG GTC ACC AAG CAC GAA AGT G-3′
NFκB	Mouse	5′-GCT GCC AAA GAA GGA CAC GAC A-3′	5′-GGC AGG CTA TTG CTC ATC ACA G-3′
NOX-5	Human	5′-GGT TTT ACC GCT CCC AGC AGA A-3′	5′-CTT CCA TGC TGA AGC CAC GCT T-3′
NOX-1	Mouse	5′-CTC CAG CCT ATC TCA TCC TGA G-3′	5′-AGT GGC AAT CAC TCC AGT AAG GC-3′
Nrf2	Human	5′-CAC ATC CAG TCA GAA ACC AGT GG-3′	5′-GGA ATG TCT GCG CCA AAA GCT G-3′
	Mouse	5′-GGC AAC AGT AGC CAC ATT GGC T-3′	5′-GTC TGG ATG GTC ATT TCA CCG C-3′
SOD	Human	5′-CTG GAC AAA CCT CAG CCC TAA C-3′	5′-AAC CTG AGC CTT GGA CAC CAA C-3′
	Mouse	5′-GGT GAA CCA GTT GTG TTG TCA GG-3′	5′-ATG AGG TCC TGC ACT GGT ACA G-3′

Table S2. Table of Antibodies.

Primary Antibody	Company	City, State in USA	Catalog no.	Recommended Concentration
ACE2	Proteintech	Rosemont, IL	21115-1-AP	
B-actin	R&D	Minneapolis, MN	MAB8929	1 mg/mL
Cl. Caspase3	R&D	Minneapolis, MN	MAB835	0.5 mg/mL
HMGB1	Invitrogen	Rockford, IL,	PA5-79373	500 μg/mL
Il-1β	NOVUS	Centennial, CO,	NBP2-27342	0.5 mg/mL
Il-6	Invitrogen	Rockford, IL,	PM626	1 mg/mL

Lycopersicon Esculentum Lectin, DyLight™ 488	Vector	Newark, CA,	DL-1174-1	1mg/mL
MyD88	Invitrogen	Rockford, IL,	PA5-19919	1 mg/mL
S-100	Novus	Centennial, CO,	NB200-538	0.5 mg/mL
TLR8	Invitrogen	Rockford, IL,	PA5-80137	500 µg/mL
TNFα	NOVUS	Centennial, CO,	NBP1-19532	1 mg/mL
TRAF6	Invitrogen	Rockford, IL,	PA1-41008	1 mg/mL

Figure S1

Glucose tolerance test.

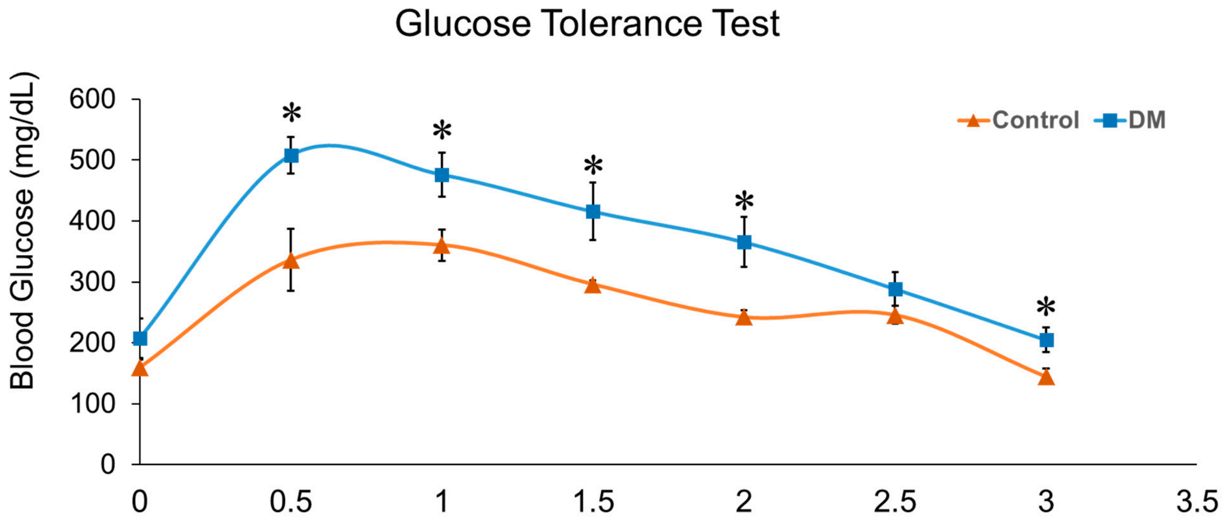

Control and diabetic hACE2 mice fasted overnight. Glucose solution (2 g/kg body weight) was administered (ip, bolus) into awake mice. Blood glucose was measured at 0.5, 1, 1.5, 2, 2.5, and 3 hrs following glucose injection. Blood glucose was blotted against time. Diabetic mice showed higher fasting glucose levels and a longer time to return to fasting levels compared to the control mice. (One-Way ANOVA, *P<0.05, n=6-8).
